# Radiotherapy in Preclinical Models of Brain Metastases: A Review and Recommendations for Future Studies

**DOI:** 10.7150/ijbs.91295

**Published:** 2024-01-01

**Authors:** Wen Shi, Guilong Tanzhu, Liu Chen, Jiaoyang Ning, Hongji Wang, Gang Xiao, Haiqin Peng, Di Jing, Huadong Liang, Jing Nie, Min Yi, Rongrong Zhou

**Affiliations:** 1 Department of Oncology, Xiangya Hospital, Central South University, Changsha, 410008, Hunan Province, China.; 2 Department of Technology, Hunan SJA Laboratory Animal Co., Ltd., Changsha, Hunan Province, China.; 3 Xiangya Lung Cancer Center, Xiangya Hospital, Central South University, Changsha, 410008, Hunan Province, China.; 4 National Clinical Research Center for Geriatric Disorders, Xiangya Hospital, Central South University, Changsha, 410008, Hunan Province, China.

**Keywords:** Brain metastasis, Radiotherapy, Dose fractionation, Radiation, Animal models, Combined modality therapy

## Abstract

Brain metastases (BMs) frequently occur in primary tumors such as lung cancer, breast cancer, and melanoma, and are associated with notably short natural survival. In addition to surgical interventions, chemotherapy, targeted therapy, and immunotherapy, radiotherapy (RT) is a crucial treatment for BM and encompasses whole-brain radiotherapy (WBRT) and stereotactic radiosurgery (SRS). Validating the efficacy and safety of treatment regimens through preclinical models is imperative for successful translation to clinical application. This not only advances fundamental research but also forms the theoretical foundation for clinical study. This review, grounded in animal models of brain metastases (AM-BM), explores the theoretical underpinnings and practical applications of radiotherapy in combination with chemotherapy, targeted therapy, immunotherapy, and emerging technologies such as nanomaterials and oxygen-containing microbubbles. Initially, we provided a concise overview of the establishment of AM-BMs. Subsequently, we summarize key RT parameters (RT mode, dose, fraction, dose rate) and their corresponding effects in AM-BMs. Finally, we present a comprehensive analysis of the current research status and future directions for combination therapy based on RT. In summary, there is presently no standardized regimen for AM-BM treatment involving RT. Further research is essential to deepen our understanding of the relationships between various parameters and their respective effects.

## Introduction

With prolonged patient survival and advancements in imaging technology, the incidence of brain metastases (BMs) is on the rise 1-4. Common primary tumors associated with BM include lung cancer 5-7, breast cancer 4,8,9, and malignant melanoma 10, 11. Despite multiple interventions, such as surgery, radiotherapy (RT), and chemotherapy, patients with brain metastases face disappointingly short survival, with a 2-year survival rate that is less than 10% 12. In recent years, the application of targeted therapy and immunotherapy has led to improvements in survival 4,12,13.

Radiotherapy (RT) is the cornerstone treatment for BM and enhances the local control rate 14 and reduces BM recurrence 15-18. The RT options for BM treatment include whole-brain radiotherapy (WBRT) 19 and stereotactic radiosurgery (SRS) 20-22. Prophylactic cranial irradiation (PCI), a unique form of RT, is known to delay and reduce the occurrence of BM 23-25. WBRT is typically recommended for patients with multiple brain metastases (usually > 3 lesions 19), while SRS may serve as the standard treatment for oligometastatic lesions (usually ≤ 4 lesions 20-22, 26). In addition to its direct impact on the BM, RT alters the tumor microenvironment and the permeability of the blood-brain barrier (BBB), laying the foundation for combination therapies 27-29. Currently, the sequence and timing of combining RT with immunotherapy 30-34, targeted therapy, or new treatments such as nanomaterials 35-38 are key research areas in the field of BM treatment.

Animal experiments play a pivotal role in preclinical research, offering a theoretical basis for clinical translation. However, distinct treatment regimens yield varied effects, and the parameters from model establishment to treatment delivery are diverse. Although RT parameters (such as RT mode, dose, dose rate, fractionation, etc.) have been explored in subcutaneous models of various tumors, these models are limited by replicating the intrinsic structure of the BBB and the unique immune microenvironment of the BM. Consequently, honest evaluations of RT and drug efficacy for treating BM are challenging. Intracranial patient-derived tumor xenograft (PDX) models, more akin to the phenotype and genotype of BM patients than subcutaneous PDX models 39,40, are crucial for assessing local curative effects and their mechanisms 13.

Currently, RT parameters in animal models of brain metastases (AM-BMs) lack standardization, and there is a dearth of reviews on this topic. This review, based on AM-BM, systematically summarizes RT regimens for BM for the first time, covering model establishment for RT implementation, and providing a reliable foundation for subsequent research. Additionally, comprehensive treatment is the primary approach for treating BM. We consolidate the schemes and molecular mechanisms of RT combined with other treatments.

## Establishment of AM-BM and the Intervention Time of RT

Various methods are employed for the establishment of AM-BMs including intracerebral 33,39,41-50, intracardiac 31,32,51-54, carotid artery 55 and tail vein injection 56,57. Few studies have explored spontaneous 58 or induced AM-BM 58,59. Mice are the most commonly used animals for AM-BM 30,38,41,43,49,56,60-65. The growth and therapeutic effects of AM-BM were monitored using *in vivo* imaging systems (IVIS) or magnetic resonance imaging (MRI), despite the relatively small volume of the BM (**Figure 2E**). The evaluation indicators for RT efficacy in AM-BM typically include tumor size and lesion size, overall survival, Ki67, Caspase-3, γH2AX expression, and so on (**Figure 2G**).

**Figure 1:**
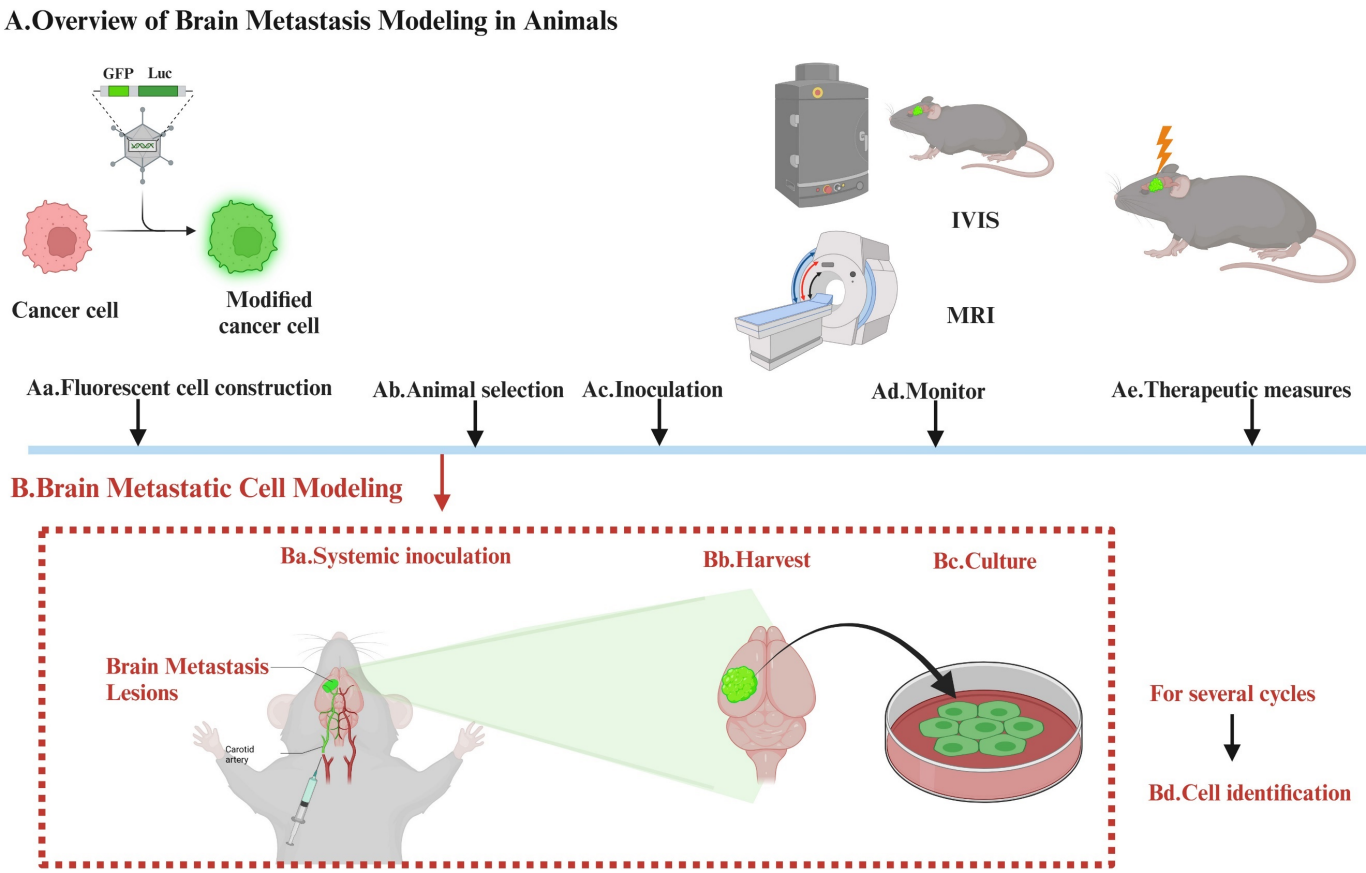
** The Process of Establishing Brain Tropic Cells.** Establishing brain-tropic cells (brain metastasis cells) requires *in vivo* and *in vitro* screening. The selection process involves modifying cancer cells with reporter genes such as luciferase or GFP, which allows changes to be easily visualized, assessed, and prepared, using IVIS or MRI. Additional rounds of selection are then carried out. The modified cells are then reintroduced into the mice, usually after a period of growth outside the body.

Establishing brain-tropic cells (brain metastasis cells) requires *in vivo* and *in vitro* screening. The selection process involves modifying cancer cells with reporter genes such as luciferase or GFP, which allows changes to be easily visualized, assessed, and prepared, using IVIS or MRI. Additional rounds of selection are then carried out. The modified cells are then reintroduced into the mice, usually after a period of growth outside the body (**Figure 1**).

**Figure 2:**
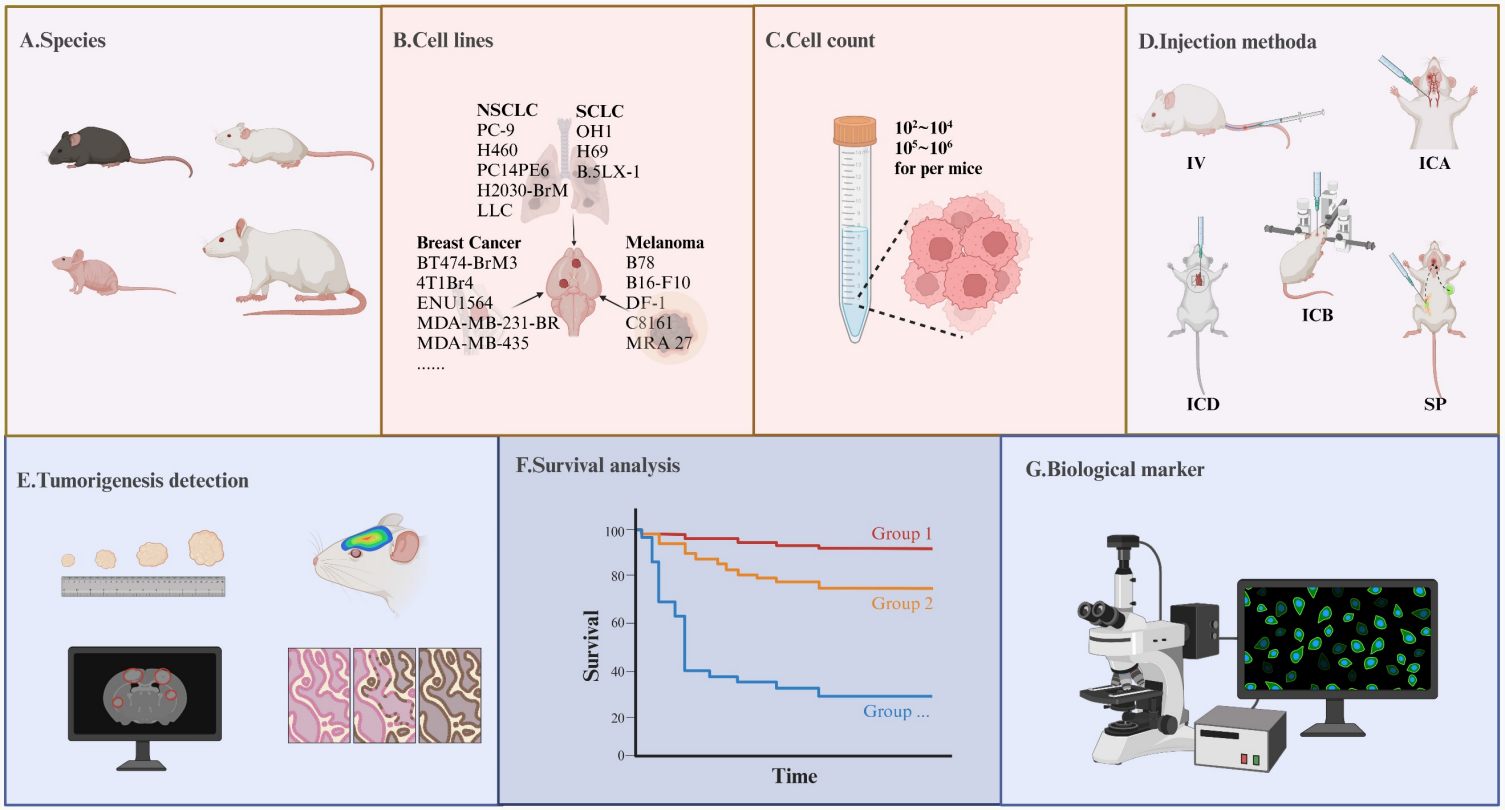
** Precautions in Model Construction and Detection Indicators in the AM-BM.** (A-D) Several key parameters significantly influence the tumor formation rate during AM-BM model development. (A) Species including C57BL/6 mice, SCID mice, BALB/c nude mice, and rats are frequently utilized in AM-BM studies. (B) Various cell lines, such as lung cancer (H2030-BrM, PC-9-BrM3), breast cancer (BT474-BrM3, 4T1Br4), and melanoma cell lines (B16-F10, B78), are commonly employed for AM-BM establishment. (C) The quantity of injected cells is a critical determinant of successful model construction and the optimal time window for treatment. (D) Current BM modeling methods encompass intracerebral injection (ICB), intracardiac injection (ICD), internal carotid artery injection (ICA), tail vein injection (IV), and spontaneous or induced models (SP). (E-G) Common parameters assessed in in-vivo studies include: (E) Tumor lesion, tumor number, and tumor volume. (F) Survival. (G) Tumor biomarkers, such as the expression of Ki67, γH2AX, and so on.

The methods for establishing AM-BMs are multifaceted, each with pros and cons. Intracerebral injection (mostly in the striatum 33,39,41-48 or cerebral cortex 49) can swiftly cause the formation of a single lesion 36, leading to high success rates. This method effectively summarizes BM growth and proliferation 66,67. However, this approach disrupts the BBB, neglects the metastasis and colonization process, and thereby weakens predictive accuracy of treatment efficacy 59. Arterial inoculation (internal carotid artery and intracardiac injection) is complex 68 and has low success rate 69,70. Due to hematogenous metastasis, the location or lesions of intracranial tumors are randomized, and the formation of multiple extracranial metastases is unavoidable 71. Intravenous (IV) inoculation (tail vein injection) is uncommon due to the low incidence of BM formation and inevitable lung metastases. Spontaneous models frequently form a single lesion in the BM 58, reflecting the actual process from tumor occurrence to metastasis. However, extensive use is hindered by the prolonged experimental period and metastases throughout the body 68. Given these considerations, we highlighted precautions for model construction and detection indicators in the AM-BM (**Figure 2**).

**Figure 3:**
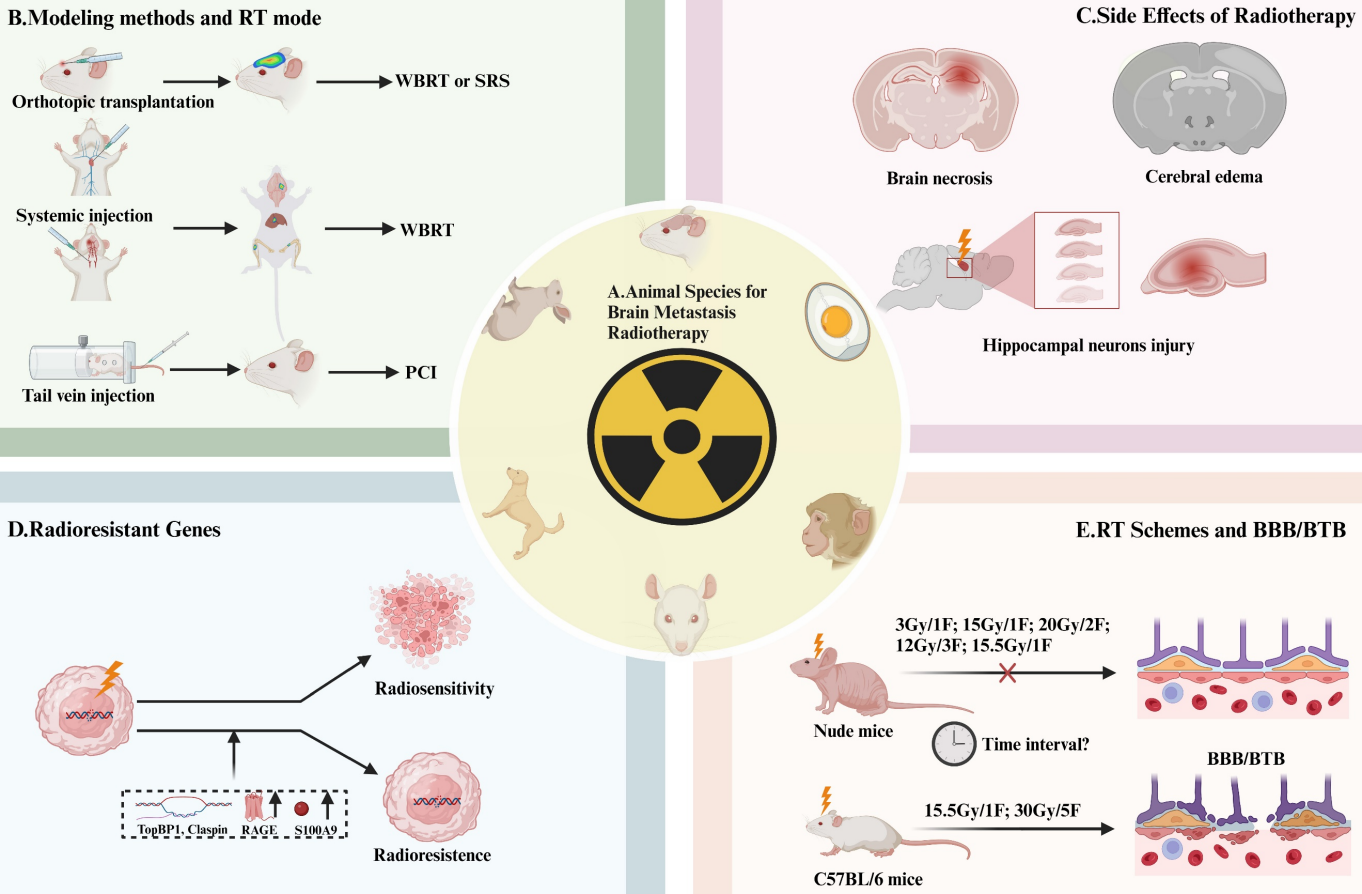
** Animal Species, Irradiation Methods Selection, and Effects of RT on AM-BM.** (A) Animal species currently employed in RT studies of AM-BM encompass mice, chicken embryos, monkeys, rats, dogs, and rabbits. (B) Different irradiation methods are utilized based on the modeling approach: SRS or WBRT is commonly applied for local inoculation modeling, WBRT for intracardiac and internal carotid artery injections, and PCI for tail vein injection. (C-D) The prognostic factors influencing survival were as follows: (C) RT-induced side effects on brain tissue, such as radiation edema, necrosis, neurotoxicity, and hippocampal damage. (D) Factors such as radiation resistance genes (TopBP1 and Claspin), secretion of S100A9, and the overexpression of RAGE limit the survival benefits of RT. (E) *In vivo* studies reveal differential responses of the blood-brain barrier/blood-tumor barrier in various mouse strains to RT. Notably, doses of 3 Gy/1F, 12 Gy/3F, 15 Gy/1F, 15.5 Gy/1F, and or 20 Gy/2F did not significantly alter the permeability of the blood-brain barrier/blood-tumor barrier in BALB/c nude mice. However, doses of 15.5 Gy/1F and 30 Gy/5F can induce changes in the blood-brain barrier/blood-tumor barrier permeability in C57BL/6 mice. The time window during which RT induces BBB/BTB opening in AM-BMs has not been determined.

The chosen modeling method influences the RT mode (**Figure 3B**). For intracerebral inoculation modeling, SRS 42,72,73 or WBRT 30,33,37,38,45,46,49,74,75 were commonly applied. Arterial inoculation-constructed AM-BM often involves WBRT 31, 32, 51-55. Additionally, PCI is used for AM-BMs established through intravenous (IV) inoculation via tail vein injection 56,57. Different model-building parameters and growth characteristics of BMs led to variable RT implementation times (**Figure 3B**).

The irradiation time of AM-BMs varies based on the inoculation methods (intracerebral earlier than systemic) and animal species (mouse earlier than rats) 48. In syngeneic models, BMs proliferate faster than in xenograft models, suggesting a shorter treatment window 68. For intracerebral injection of lung/breast cancer AM-BM, irradiation time is closely tied to injection cell number, generally, beginning within 2 weeks for 10^6^ cells 46,76 and within 2-3 weeks for 10^4^ cells 45,75,77. In melanoma AM-BM, irradiation of 10^2^-10^4^ cells usually occurs within 1-2 weeks 33,34,42,48,73. In particular, PCI is administered within one week of cell inoculation 56,57,78. The relationship between the implanted cell counts and RT intervention time is shown in **Table 1**.

## Dose and Fractionation of RT in AM-BM

RT is a conventional therapy for BM 17. However, the diversity of RT regimens in AM-BM across multiple studies underscores the necessity for standardization. Currently, WBRT is widely applied in AM-BM 30,33,34,37,38,45,46,49,54,74-77,79, followed by SRS 42,72,73. Various parameters influence RT efficacy, including the RT method, dose, fractionation, dose rate, and intervention time. A comprehensive summary of these parameters was obtained from available radiotherapy studies in AM-BM (**Table 2-6**). Additionally, a comprehensive and scientific template for reporting experiments involving AM-BM and RT is shown in** Table 7**.

The linear-quadratic (L-Q) model, typically used to calculate the biologically effective dose (BED) of different fractionation schemes employs the formula BED = D [1 + d/(α/β)] 80. The alpha/beta ratio(α/β), total dose (D), and fractional dose (d) are integral components 81. The criteria for RT schemes in AM-BM include (1) clinical regimens 62,74,76, (2) BED equivalent schemes 33,34,38,45,54,75,76,79, (3) previous experience based on different research objects 35-37,50,52,56, and (4) protection of normal tissues 49,55,82. The first two criteria are generally applied.

### WBRT

WBRT is widely employed in AM-BMs. The 30 Gy/10F or 20 Gy/5F regimens 83 are recommended by the National Comprehensive Cancer Network (NCCN) guidelines for BM patients 84, while preclinical studies typically administer WBRT at a total dose ranging from 15 Gy to 20 Gy in a single or fractionated high-dose irradiation format 33,34,38,45,54,75,76,79.

As indicated by previous reports, lower doses (< 15 Gy) of WBRT have been explored due to their ability to inhibit tumors and prolong survival 36,37,52,56. Notably, a study implementing WBRT (12 Gy/3F) significantly restricted tumor volume but failed to reduce the number of BM lesions 52. In AM-BM of breast cancer, Choi et al. demonstrated that 10 Gy/1F exhibited a stronger inhibitory effect than 5 Gy/1F, with no significant difference observed with 20 Gy/1F 65. Compared with those treated with 5 Gy/1F, 15 Gy/1F, or 20 Gy/1F, animals treated with BM via the 10 Gy/1F regimen had the longest survival 50. In combination therapy, the use of WBRT (7 Gy/1F) with nanoparticles for AM-BM of melanoma demonstrated a reduced RT dose and prolonged survival 36.

Using the L-Q model, the BED of RT regimens (15-16 Gy/1F, 20 Gy/2F) in AM-BM was found to be comparable to the clinical regimens of 30 Gy/10F, assuming the α/β value of 10 54,76,78,79. Zarghami et al. and Murrell et al. employed 16 Gy/1F and 20 Gy/2F, respectively, and showed significant reductions in BM lesions and tumor volume 54, 79. Due to the limited access to synchrotron radiation sources, some studies have applied single-dose fraction RT 85, which saves time but may increase the risk of edema and necrosis.

To mitigate side effects, certain studies have adopted regimens with lower BED for normal tissue 49,55,82. Martínez-Aranda et al. demonstrated that the 16.5 Gy/3F protocol 55, which involves a BED lower than 30 Gy/10F, significantly alleviated brain toxicity and reduced the frequency of intraperitoneal anesthesia, partially circumventing accidental death 55. Similarly, Prociss et al. verified that the 10 Gy/5F regimen effectively avoided neurotoxicity in AM-BM 49. To overcome radiation resistance in melanoma and prevent radiation necrosis simultaneously, Wall et al. raised the single dose to evaluate the effect of RT (12 Gy/3F) 82.

Alterations in the tumor microenvironment following radiation exposure also led to varied effects on tumor eradication. Different RT fractions with equivalent doses led to diverse responses of immune cells 31,32. Schulz et al. reported that compared with that of 10 Gy/1F, 10 Gy/5F increased monocyte-derived macrophage (TAM-MDM) infiltration. However, the former regimens reduced TAM-MDMs and, more significantly, altered the expression of genes related to pro-inflammatory host defense responses in peripheral myeloid cells 32. Distinct RT fractions with the same BED generated diverse inhibitory effects. For instance, 30 Gy/10F significantly reduced intracranial metastases, while 15 Gy/1F was more beneficial for median survival 76.

RT was found to delay the progression of BM and prolong survival in various studies 33,36,38,45,55,76,77,86. Hofland et al. demonstrated that in AM-BM of Ehrlich's ascites cancer, RT significantly prolonged survival, with the 10 Gy/1F regimen showing more apparent effects 50. In AM-BM of breast cancer, RT may achieve long-term survival 76. However, individual studies reported conflicting conclusions 44,49,75,87. For instance, Chae et al. showed that WBRT (10 Gy/1F) reduced tumor volume but had no significant effect on survival 53. Similarly, regimens such as 30 Gy/10F, 16.5 Gy/3F, and 9 Gy/3F did not improve tumor progression and survival due to radiation resistance 88.

**Figure 4:**
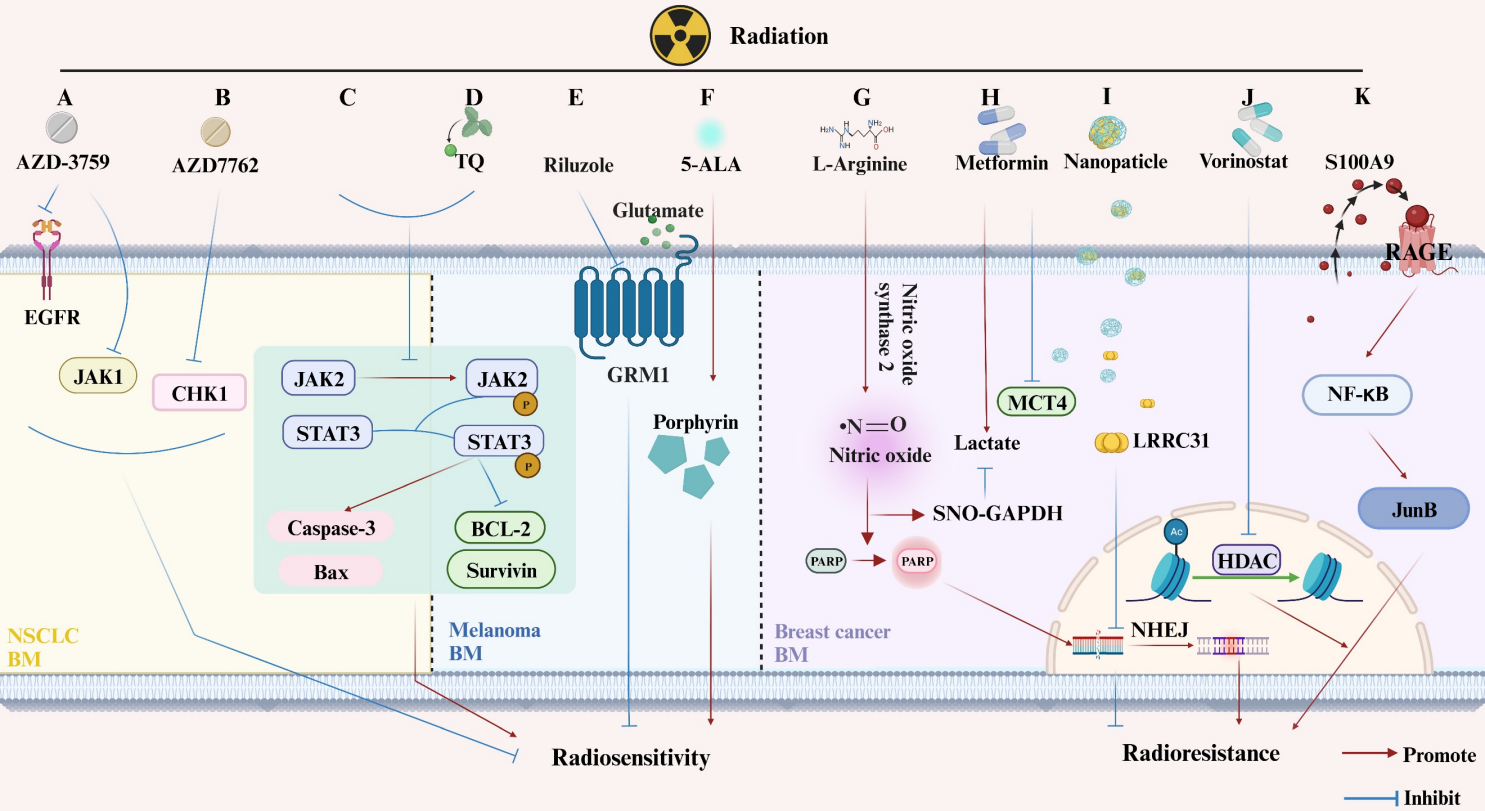
** Changes in Molecular Pathway Induced by RT with or without Additional Treatments in Animal Models of Brain Metastases from Lung Cancer, Melanoma, and Breast Cancer.** (A-C) Lung Cancer BM. (A) AZD-3759 enhances NSCLC radiosensitivity by inhibiting EGFR and JAK1; (B) AZD7762 promotes NSCLC radiosensitivity by suppressing CHK1; (C) RT inhibits phosphorylation of JAK2 and STAT3, inducing apoptosis. (D-F) Melanoma BM. (D) Thymoquinone (TQ) increases radiation-induced apoptosis by inhibiting JAK2 phosphorylation; (E) Riluzole enhances radiosensitivity of melanoma BM cells by inhabiting GRM1; (F) 5-ALA increases melanoma BM radiosensitivity by increasing the porphyrin content. (G-K) Breast Cancer BM. (G) L-arginine mediates radiosensitivity through NO-dependent inhibition of GAPDH and PARP activation; (H) Metformin enhances tumor suppression when used as an adjuvant in RT; (I) LRRC31 importation via nanomaterials inhibits DNA repair and radiosensitivity in breast cancer BM; (J) Vorinostat, a histone deacetylase inhibitor, increases radiation sensitivity by inhibiting HDAC; (K) BM secreting S100A9, which binds to the RT-induced RAGE receptor, activates NF-κB-mediated RT resistance.

Reasons for Conflicting Results in WBRT Research in AM-BM:

(1) Impact of Model Establishment on Survival. Some studies suggest that the mode of model establishment may influence survival. Arterial inoculation, which can lead to widespread tumor metastasis, raises the concern that animal death may not be solely attributed to BM 76.

(2) Side Effects Caused by RT. The side effects of RT such as radiation edema, necrosis, nerve damage, and hippocampal damage can vary and impact study outcomes. WBRT (10-15 Gy) has been observed to inhibit nerve growth by inducing DNA double-strand breaks (DSBs) and apoptosis 53,76,89. Additionally, single high-dose RT may lead to hippocampal toxicity 76,90 (**Figure 3C**).

(3) Inconsistent Radiosensitivity among Various Cancer and Cell Subtypes. The calculation of the biologically effective dose (BED) by the linear-quadratic (L-Q) model may be inaccurate, especially after standardizing the empirical rule α/β=10 across various tumors 91. Moreover, the L-Q model is not suitable for relatively high doses (>13 Gy) 92.

(4) Radiation-Resistant Genes. The presence of radiation-resistant genes, such as TopBP1, Claspin, and Caveolin 1, in AM-BM of NSCLC, may influence the effectiveness of RT (**Figure 3D**). These genes alleviate inhibition, leading to improved survival following RT 75,93. Furthermore, the expression and secretion of S100A9 from BM cells, which binds to the RT-induced RAGE receptor, activate NF-κB mediated RT resistance 88 (**Figure 4K**).

In conclusion, these factors highlight the complexity of interpreting and comparing results across different studies. A summary of the relevant parameters for WBRT in AM-BM is provided in **Table 2 and Table 3**.

### SRS

SRS, which is commonly administered to single lesions 35,42,72,73 in AM-BM, has received limited research attention. The dosage of SRS tends to decrease when combined with other treatments 35,42. Numerous studies have demonstrated that SRS contributes to prolonged survival 42,72,73. Notably, Nakahara et al. recently reported a significant increase in survival when SRS (32 Gy/1F) was combined with immunotherapy 72. Additionally, they observed the inhibition of JAK2 and STAT3 phosphorylation after SRS (15 Gy/1F). This inhibition, in turn, triggered cell death by regulating apoptosis-related proteins, such as increased Caspase-3 and BAX and decreased BCL-2 and Survivin 42 (**Figure 4C**). A summary of relevant parameters from SRS studies in AM-BM is presented in **Table 4**.

### PCI

In contrast to therapeutic irradiation, the primary objective of PCI is to reduce the incidence of BM 25. This perspective is supported by various preclinical studies and computer models 56,57,78. In preclinical studies targeting breast cancer, 4 Gy/1F at 3.2 Gy/min and 20 Gy/2F significantly lowered the occurrence of BM 56,78. However, eliminating dormant cells in BM has shown to be challenging with PCI, which accounts for subsequent tumor occurrence 78.

A critical consideration involves determining the optimal timing for PCI intervention in AM-BM. Studies demonstrate that performing PCI within 1-5 days after tumor cell injection 56,57,78 consistently reduces the incidence of BM. In contrast, executing PCI either before or 3-6 weeks after systemic inoculation poses challenges and may not achieve optimal outcomes 56. Premature PCI interventions may even promote tumor progression and metastatic formation by inducing alterations in the brain microenvironment. For instance, administering RT (10 Gy/1F) seven days before injection results in damage to normal brain tissue, which becomes more susceptible to the growth of BM 51. The relevant parameters of the PCI research are detailed in **Table 5**. Moreover, for those that do not specify the specific radiotherapy method, the parameters are detailed in **Table 6**.

## RT and the BBB/BTB

Both clinical and preclinical studies have consistently demonstrated that RT induces an increase in the permeability of the BBB or blood-tumor barrier (BTB), resulting in elevated intracranial drug concentrations. These findings form the theoretical basis for combination therapy 27-29,94-98. In addition, RT (30 Gy/5F) combined with focused ultrasound (FUS) disrupts BBB integrity 99. Interestingly, a subset of in-vivo studies has reported contradictory conclusions. Notably, high-dose irradiation whether delivered as a single dose or in a fractionated manner, has shown limited impact on BBB/BTB permeability in certain scenarios 52,54. In AM-BM of lung/breast cancer (nude mouse models), after 3 Gy/1F 74, 12 Gy/3F 52, 15 Gy/1F 74, 15.5 Gy/1F 100, or 20 Gy/2F 54 irradiation did not significantly alter BBB permeability and, in some cases, even led to a short-term decrease (within 24 hours) 74. The immune function of the BM model may contribute to this observed phenomenon 100. For instance, twelve hours after 15.5 Gy/1F irradiation, changes in the integrity of the BBB and the activity of efflux transporters were noted in immunocompetent mice, while no such differences were observed in immunocompromised mice. This finding implies a potential association between the immune response and BBB damage after RT 100. It is essential to consider the time interval during which RT induces BBB opening, and variations in sensitivity among different detection methods should be taken into account (**Figure 3E**).

## Combination therapy in AM-BM

### RT Combined with Chemotherapy

Chemotherapy has been shown to augment the radiosensitivity of BM 50,55,101,102. Temozolomide (TMZ) 55,102, etoposide 50, and dexrazoxane 50, when combined with RT, effectively inhibit the progression of AM-BM. TMZ, recognized for its ability to penetrate the blood-brain barrier, is recommended as a chemotherapeutic drug for intracranial tumors 103. Furthermore, TMZ enhances the radiosensitivity of brain metastatic tumor cells by inhibiting DNA damage repair after RT and amplifying mitotic catastrophe 102. RT combined with TMZ has been shown to prolong survival in AM-BM of breast cancer 55. Meanwhile, in the AM-BMs of NSCLC, non-ablative radiation (2 Gy) enhances the delivery of anti-MGMT morpholino oligonucleotides (AMONs) improving TMZ efficacy by inhibiting MGMT 43. Etoposide plus dexrazoxane, combined with WBRT (10 Gy/1F) increased the median survival by 60% with no additional toxicity 50. Similarly, an antibody-drug conjugate such as BR96-DOX in combination with RT significantly prolonged survival in AM-BMs of SCLC 77. Furthermore, compared with concurrent chemoradiotherapy, antibody-drug conjugates administered before RT improved survival 77.

### RT Combined with Targeted Therapy

The combination of RT and targeted therapy typically has synergistic effects 104. WBRT enhances the therapeutic effect of single domain antibody fragment (Anti-HER2 VHH 5F7) on human epidermal growth factor receptor type 2 (HER2) positive BM by increasing vascular permeability 49. Overall, targeted c-Met and RT inhibit tumors and prolong the overall survival of tumor-bearing mice 44.

Similarly, targeted agents enhance the efficacy of radiotherapy. Targeting EGFR 62, CHK1 45, HDAC 47, CXCR4 71, ATR 47, and GRM1 82 significantly improved RT efficacy. For instance, AZD3759 (zorifertinib) amplifies the antitumor effect of RT by interfering with EGFR and JAK1 62 (**Figure 4A**). AZD7762 enhances radiosensitivity *in vitro* and *in vivo* by inhibiting CHK1 45 (**Figure 4B**). Vorinostat improves the median survival of AM-BMs by blocking histone deacetylases (HDACs), leading to DSBs repair inhibition and mitotic catastrophe 47 (**Figure 4J**). Riluzole (a glutamate signaling blockade) sensitized cells to RT (**Figure 4E**). Mechanistically, inhibition of glutamate signaling led to G2/M phase arrest in melanoma cells 82.

Additionally, targeted drugs modulate the tumor microenvironment 71,82. Endostar enhances RT efficacy by blocking RT-induced CXCR4. Subsequently, TAM infiltration and macrophage M2 polarization are inhibited, and the percentage of CD4+T/CD3+T cells increases 71.

### RT Combined with Immunotherapy

Immunotherapeutic strategies, including immune checkpoint inhibition (ICI), adoptive cells, tumor vaccines, oncolytic viruses, and cytokine therapy, are integral components of AM-BM treatment. Currently, the integration of immunotherapy with RT in AM-BM primarily involves ICI and *in*-*situ* vaccination (ISV), both of which enhance RT efficacy. Notably, combining a tumor vaccine with RT (15 Gy) significantly reduces tumor volume and delays BM progression 33.

RT contributes to immunotherapy efficacy by regulating the tumor microenvironment 30-32. WBRT recruits myeloid cells and enhances their proinflammatory response 32. Meanwhile, WBRT significantly elevated TNF-α and CXCL1 in the serum of immunocompetent and immunocompromised mice 100. The levels of proinflammatory cytokines (TNF-α, IL-2, and IL-12p70) increase after WBRT in immunocompetent mice but not in nude mice 100. Furthermore, increasing the radiation dose (from 15 Gy to 18.5 Gy) improved immunotherapy efficacy in AM-BM, resulting in longer survival and tumor dormancy periods 34. Relatively low-dose WBRT (4 Gy) or targeted radionuclide therapy increased the number of T cells (CD4+ and CD8+) and the monocyte/macrocytic phagocytic (F4/80+) population, enhancing the immunotherapy response in melanoma BM 30.

The sequencing of immunotherapy and RT needs to be further explored. Transcriptome analysis revealed that RT following ICI treatment is involved in cell death and inflammation signaling in melanoma BM. Preclinical studies have demonstrated that RT followed by anti-PD-L1 therapy is preferable 105, which has also been confirmed in clinical trials 106, 107.

### Combination of RT and Novel Technologies

Ongoing exploration by radiation biologists has led to the application of novel technologies to AM-BM. Compared with conventional RT, FLASH radiotherapy (FLASH-RT) and heavy ion radiotherapy exhibit superior curative effects with relatively fewer adverse events 108, holding promise for BM treatment 109.

The optimization of drug carriers has also advanced the field of BMs treatment. RT combined with ultrasound-mediated rupture of oxygen-carrying microbubbles (MBs) delays tumor progression and improves survival in AM-BM of breast cancer 86. In addition, nanoparticles enhance the therapeutic effect of RT on BM by modulating radiosensitivity 35-37 or increasing the dose absorption of RT 38 (**Figure 4I**).

The development of novel medicines is equally compelling for researchers. 5-aminolevulinic acid (5-ALA), a novel photodynamic drug, enhances the radiosensitivity of melanoma BM by upregulating protoporphyrin IX (PpIX) 101 (**Figure 4F**). Thymoquinone (TQ) improves the efficacy of gamma knife therapy on melanoma BMs, by enhancing apoptosis through regulation of the JAK2/STAT3 pathway 42. Moreover, TQ induces the secretion of inflammatory growth factors 42 (**Figure 4D**). Metformin increases the concentration of lactate in MDA-MB‐231-Br cells by suppressing the MCT4 protein, thereby enhancing the anti-tumor effect of RT 65 (**Figure 4H**). In the AM-BM of breast cancer, L-arginine amplifies RT efficacy by modulating nitric oxide metabolism 110 (**Figure 4G**). Additionally, magnetic field therapy (athermal radiofrequency electromagnetic fields) combined with RT significantly inhabits radiation-resistant cells and prolongs the survival of AM-BMs 111.

## Future and Prospects

The two-year survival rate for patients with BM is typically less than 10% 12. RT, including WBRT and SRS, is one of the essential treatments for BM. The topic of brain metastases has attracted much attention in the 2023 oncology conferences (such as ASCO, ASTRO, and WCLC). Dose exploration remains a key topic, particularly in the field of radiotherapy for brain metastases. This review, based on the AM-BM of various tumors, presents a comprehensive summary of preclinical research on BM and RT for the first time. We focused on RT parameters, including modality, total dose, fractionation, dose rate, and their corresponding effects. Additionally, we highlight recent advancements in the study of BM with RT, emphasizing combination with chemotherapy, targeted therapy, immunotherapy, and novel technologies.

Animal models for BM encompass diverse species, including mice 30,38,41,43,49,56,60-65, rats 48,72,77,112, 113, monkeys 114, dogs 115, rabbits 116, and chick embryos 117-119, with mice being predominantly the predominant ones used (**Figure 3A**). The organ tropism of tumor cells in chick embryos was recently found to be consistent with that in mice 118. The location of the BM in most studies is limited to the cerebral cortex 49, 50 or the striatum 33, 39, 41-48. Establishing the AM-BMs in specific anatomical locations, such as the leptomeninges 120, 121 and cerebellum 122, requires further exploration. The "seed" and "soil" interactions during tumor metastasis endow the primary tumors and metastases with different characteristics, the selection and establishment of brain-tropic cells necessitate attention 68. Radiation-resistant models have also received limited research attention. In recent years, the application of humanized mice, microfluidic chips mimicking 123, 124, PDX models 39,40, and organoids 125 has emerged, enhancing the translatability of research in the field of BM. The application of emerging models, diagnostic methods, and treatment techniques to study brain metastasis may catalyze its clinical transformation and change treatment paradigms, which deserves further attention. Single-cell sequence and spatial transcriptomics offer promising avenues for obtaining more authentic information about BMs. Meanwhile, the difference in the organ affinity of primary tumor metastases to brain tissue needs to be further explored. Owing to the rarity of brain metastatic cells, certain studies have resorted to employing cell lines derived from primary tumors to investigate the relevant mechanisms involved. Although validated in animal models, the exploration of brain metastasis mechanisms outside the brain microenvironment has somewhat compromised the persuasiveness of the conclusions. This issue is currently a focus in the field of brain metastasis research. Continuous optimization of animal model construction and the development of emerging models may offer a potential solution.

For clinical transformation, preclinical studies have mostly used mice, which have certain differences in genetics, radiation sensitivity, and other aspects compared to humans. Like the parameters of chemotherapy and immunotherapy, preclinical radiation dosimetry parameters are difficult to convert and apply to humans. In terms of dosage, preclinical models can only provide positive (effective tumor suppression) or negative (failure to tumor suppression) results, which is an unavoidable problem in clinical transformation. Utilizing and optimizing models with personalized patient information, such as PDX models and organoids, is more convincing and easier to use for achieving clinical conversion.

The choice of the RT method is influenced by the establishment of animal models. BM formed by intracerebral inoculation is commonly treated with WBRT or SRS, whereas intracardiac and carotid artery injections (systemic injection) may generate multiple intracranial metastases, often treated with WBRT in AM-BM. In addition, intravenous injection is relatively more frequently used in PCI research. However, whether the carotid artery or intracardiac injection is suitable for PCI research still needs convincing evidence.

The timing of RT intervention is also worthy of attention. The AM-BMs of breast/lung cancer (10^6^ tumor cells) or melanoma (10^4^ tumor cells) constructed by intracerebral injection are generally administrated RT within two weeks. Meanwhile, for systemic inoculation, the irradiation time for intracardiac injection modeling is generally later than that for intracerebral transplantation. Notably, the timing of RT intervention and the definition of the irradiation field between AM-BM and clinical application pose challenges but advances in animal imaging technology and RT may provide solutions.

Current RT regimens for AM-BM include four criteria: (1) clinical regimens 62,74,76, (2) BED equivalent regimens 33,34,38,45,54,75,76,79, (3) experience-based regimens 35-37,52,56, and (4) protection of normal tissues 49,55,82. Dose and fractionation emerge as critical factors. Presently, BED equivalent schemes (based on the L-Q model) are predominantly applied. Most studies employ either single high-dose irradiation or high-dose fractional irradiation (15-16 Gy/1F) 33,34,38,45,54,75,76,79. However, some researchers argue that the L-Q model is unsuitable for high-dose irradiation 126, in which the dose-survival curve of tumor cells is significantly shifted, accounting for decreased predictive accuracy 81,92. Meanwhile, for BED calculations, α/β is not entirely consistent across different tumors and cell subtypes 91. While RT alone or combined with other treatments effectively inhibits BM, inevitable adverse events, including radiation necrosis, cerebral edema, and neuronal damage, can occur 127. Due to the substantial heterogeneity among various studies, drawing definitive conclusions regarding optimal RT regimens or combination therapies for maximum benefit is challenging.

Owing to the limited volume of BM in animals, only a few preclinical studies have reported IMRT in AM-BM 86,128. Conformal magnetic resonance imaging (MRI) in rats contributes to precise RT to some extent 112. Currently, the Small Animal Radiation Research Platform (SARRP) (Xstrahl, Camberley, UK) 129,130, X-RAD SmART (Precision X-ray, North Branford, Connecticut, USA) 131, and the SAIGRT system 132 have been validated to achieve small-volume precise radiotherapy in AM-BM. Moreover, Delaney et al. conducted IMRT for mice BMs using SARRP combined with cone-beam computed tomography guidance 86. Interestingly, they demonstrated that the clinical linear accelerator Novalis TX (Brainlab AG, Feldkirchen, Germany) could also achieve IMRT in AM-BM 128.

**Figure 5:**
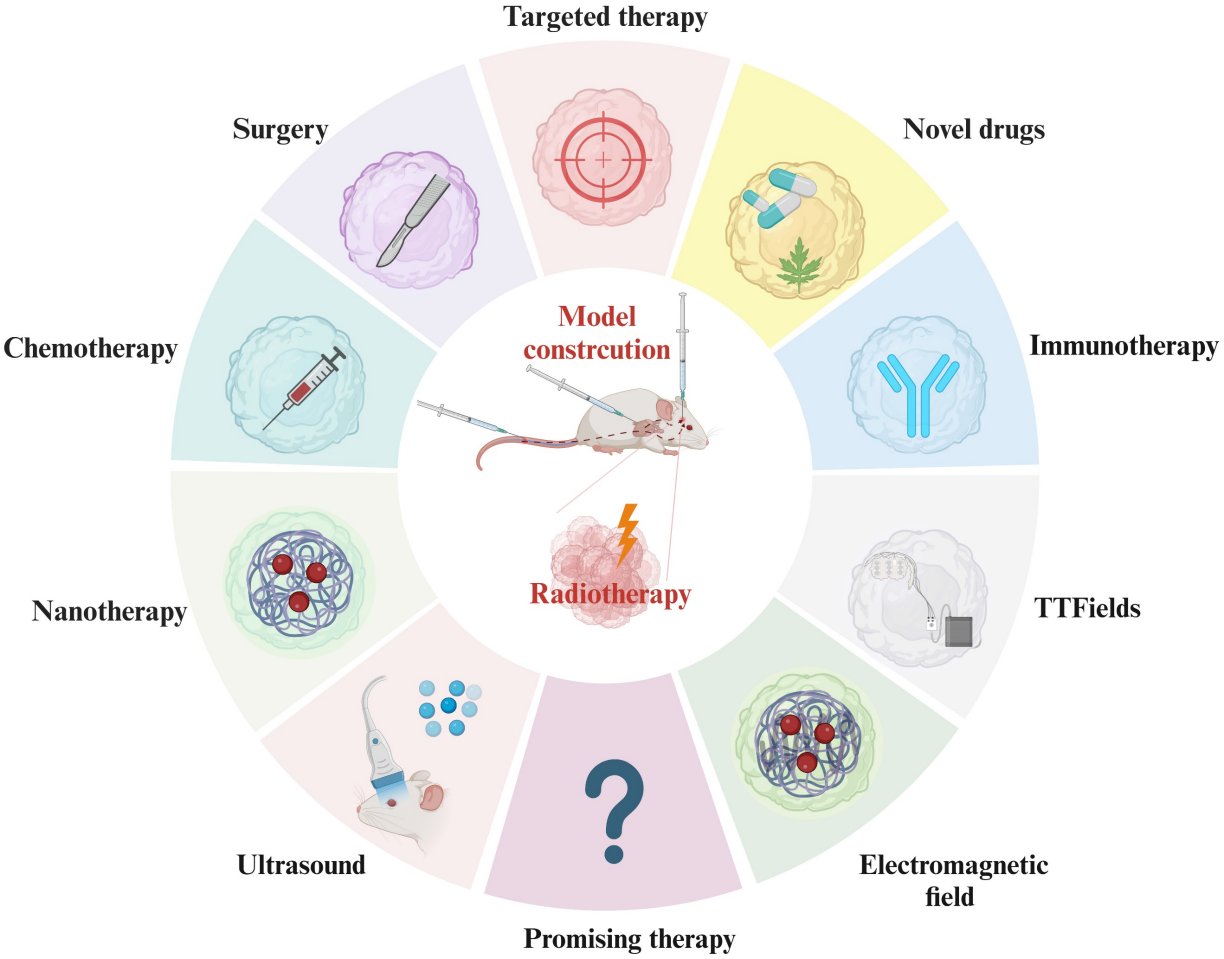
** Combination Treatment with RT in Current AM-BM Research.** Current research on AM-BMs explores diverse combination treatments with RT, including immunotherapy, novel drug applications, targeted therapy, surgery, chemotherapy, nanomaterial applications, ultrasound (mediating oxygen-containing microbubble rupture), magnetic field therapy, and electric field therapy. These comprehensive approaches signify the multifaceted strategies being investigated to optimize the efficacy of RT in AM-BM.

Innovative irradiation methods such as HA-WBRT, FLASH-RT 108, and heavy iron therapy 109, may yield improved therapeutic effects in BM. Recent studies have explored tumor-treating fields 133 and athermal radiofrequency electromagnetic field 111. Targeted therapy, immunotherapy, and novel technologies like nanoparticles and oxygen-containing microbubbles have been extensively studied in primary tumors, but their exploration in metastatic tumors is limited (**Figure 5**). Tumor cells evolve during metastatic periods, and the characteristics of metastatic lesions are not consistent with those of primary tumors. Moreover, the BBB establishes a particular intracranial immune environment. Consequently, the application of novel treatment methods and technologies for treating BM warrants further investigation. The mechanisms by which RT modulates the BBB and regulates the microenvironment of the BM demand in-depth exploration.

In conclusion, the choice of RT regimens in BM depends on the model establishment. It is imperative to focus on refining RT or comprehensive treatment protocols for AM-BM and strive for the standardization of preclinical research on RT to facilitate its clinical application. However, further studies are needed to elucidate how to optimize the efficacy of RT in BM.

## Figures and Tables

**Table 1:** Cells Account and RT Intervention Schedule for Intracerebral Injection (Part 1) and Other Injections (Part 2) (including internal carotid artery and intracardiac injection).

**Part 1**			
**Cancer type and cell count**	**0-1 Weeks**	**1-2 Weeks**	**2-3 Weeks**
**Lung cancer**			
**10^3^-10^4^**	NA	1×10^4^ (WBRT, PC14PE6) 45, 75	NA
**10^4^-10^5^**	NA	1×10^5^ (WBRT, LX-1) 77	1×10^5^ (WBRT, LLC) 71
**10^5^-10^6^**	2×10^5^ (WBRT, PD*) 46	NA	1×10^6^(conformal RT, H460) 43
**Breast cancer**			
**10^3^-10^4^**	5×10^3^ (SRS, MADB106) 72	NA	1×10^3^ (IMRT, ENU1564) 112
**10^4^-10^5^**	2×10^4^(WBRT, MDA-231-Br) 37; 2.5×10^4^(WBRT, E0771-BrM3) 88	NA	1×10^5^ (WBRT, MDA-MB-435) 44
**10^5^-10^6^**	NA	1×10^6^ (WBRT, MDA-231-Br) 76; 4×10^5^ (WBRT, BT474-Br-M3) 77	1.75×10^5^ (WBRT, MDA-231-Br) 87
**Melanoma**			
**10^2^-10^3^**	3×10^2^ (SRS, B16-F10) 73; 5×10^2^ (SRS, B16-F10) 42	NA	NA
**10^3^-10^4^**	NA	2×10^3^ (WBRT, B16) 33; 5×10^3^ (WBRT, B16-F10) 34	NA
**10^5^-10^6^**	NA	1×10^6^ (IR*, MRA 27) 48	NA
**Ehrlich ascites tumor**			
**10^5^-10^6^**	1.5×10^5^ (WBRT, Ehrlich ascites tumor cells) 50	NA	NA
**Part 2**			
**Cancer type and cell count**	**0-1 Weeks**	**2-3 Weeks**	**3-4 Weeks**
**Lung cancer**			
**10^5^-10^6^ cells**	1×10^5^ (WBRT, H2030-BrM; intracardiac injection) 88	2×10^5^ (WBRT, LLC; intracarotid artery) 71	NA
**Breast cancer**			
**10^4^-10^5^ cells**	NA	3×10^4^ (WBRT, TS1-BrM; intracardiac injection) 31; 1×10^5^ (WBRT, 99LN-BrM; intracardiac injection) 31	NA
**10^5^-10^6^ cells**	1.75×10^5^ (PCI, MDA-231-Br-HER2; intracardiac injection) 78 5×10^5^ (PCI, MDA-IBC-3; tail vein injection) 56	1.75×10^5^ (WBRT, C8161; intracardiac injection) 76; 2×10^5^ (WBRT, MDA-231-Br; intracardiac injection) 52;	1×10^5^ (WBRT, MDA-231-Br; intracardiac injection) 54; 1.5×10^5^ (half brain radiation, MDA-231-Br; intracardiac injection) 79; 1×10^6^ (WBRT, 435-Br1; intracarotid artery) 55

*PD: patient-derived tissue cells; IR: No specific radiotherapy method was mentioned.

**Table 2:** Relevant Parameters of Radiotherapy Research in the AM-BM (WBRT combined CTR, IT, TT, etc.)

Cancer	Species	Monitoring intracranial tumor formation	Injection method	Injection site	Cell line	Cell number/volume (cells/μl)	Irradiator	Irradiation time (days)	Dose/fraction (Gy/F)	Dose rate (Gy/min)	Combination therapy	Effect	Ref
**NSCLC**	8w Male athymic nude mice	NA	ICB	Left striatum	PC14PE6 (used modeling) H23	1×10^4^/5	IBL 437C blood Irradiator	14	15/1	2.3	TT: Chk1 AZD7762	Median Survival RT ↑ RT+AZD7762 ↑↑	45
**NSCLC**	Female nude (rnu/rnu) rats	Fluorescent oligonucleotide delivery	ICB	Right caudate nucleus	H460	1×10^6^/10	RadSource RS2000 Irradiator Versa HD (Elekta, Stockholm, Sweden) linear accelerator	21 (D283) 5 (H460)	2/1 (athymic rat) 5/1 (normal Long Evan rats)	NA	CTR+TT: TMZ (20 mg/kg × 4 days) anti-MGMT morpholino oligonucleotides (AMONs) (10.5 mg/kg; IV) (1 d after radiation)	(vs. RT+TMZ) Tumor volume RT+TMZ+AMON ↓ (RT+AMON vs RT) MGMT — BCL-XL — p27 —	43
**NSCLC**	7-8w BALB/c nude mice	Detecting fluorescence intensity.	ICD	NA	PC-9 (modeling) H3255	NA/100	XCELL 160 X-ray system (Kubtec, Stratford, CT, USA)	intracranial fluorescent signal >5×10^6^ photons/s (1-3W)	30/10	1.6	TT: EGFR AZD3759 or osimertinib (1 h prior to the RT)	Tumor volumes RT ↓ RT + AZD3759 ↓↓↓ RT + osimertinib ↓↓	62
**NSCLC**	6-8w Female nu/nu mice	Bioluminescence signals	ICB	Brain parenchyma	PC-9 (modeling) H3255 H2228 H226	5×10^5^/NA	XCELL 160 X-ray system	NA	30/10 15/1 3/1	1.6	TT: EGFR 1 h before radiation. (AZD3759 was administered by oral gavage at 15 mg/kg once daily until the end of the study)	*In vivo* (vs RT) Tumor volume RT+AZD3759↓↓ Ki-67 RT↑ (3h ) — (8h) RT+AZD3759↓↓ CC3 RT↑ RT+AZD3759↑↑	74
**NSCLC**	6-8w Female athymic Nude-Foxn1nu mice	IVIS	ICB	Right striatum	PDX*	2×10^5^/5	Xstrahl Small Animal Radiation Research Platform (SARRP)	3	12.5/5	2.68	TT: ATR M6620(60 mg/kg by daily oral gavage)	M6620 treatment combined with radiotherapy synergistically and successfully inhibits cancer growth (PDX)	46
**NSCLC**	6-8w Female C57BL mice	IVIS 1. 14 days 2. 6 days	1. ICA 2. ICB	Right striatum	LLC	1. 2×10^5^/100 2. 1×10^5^/5	Varian Clinac 600C X-ray unit	1.21 2.13	10/1	2.5	TT: CXCR4 Endostar (ES) (Treatment 14 days after IVIS imaging)	*In vivo* Tumor size RT↓ RT + Endostar↓↓ The vessels: RT + Endostar more regular and pericyte	71
**Melanoma**	6-7w athymic nude mice	IVIS	ICB	0.5 mm anterior and 2 mm lateral to the bregma	C8161	1×10^4^/5 (0.6 μL /min)	γ-irradiator (Gammator 50, cesium 137 source)	NA	16/4/4 weeks	NA	TT: GRM1 Riluzole	Tumor volume RT ↓ RT + Riluzole ↓↓	82
**Melanoma**	6-8w Female C57BL/6 mice	NA	ICB	Right striatum	B78	2×10^5^/NA	X-RAD 320 (Precision X-Ray Inc., North Branford, CT)	Day 1 after flank irradiation or day 15	4/1	NA	IT: ISV (1 day) + anti-CTLA-4(3,6,9 days)	Relatively low-dose WBRT (4 Gy) or targeted radionuclide therapy increases the number of T lymphocytes (CD4+ and CD8+) and monocytes/macrophages (F4/80+) and improves immunotherapy of bone marrow melanoma reaction.	30
**Melanoma**	6w C57BL/6J mice	MRI	ICB	NA	B16-F10	5×10^4^/NA	320 kV X-ray generator	NA	7/1	2	AGuIX® (Gd-based nanoparticles) (10 mg, i.v.) (radiation 3.5 hours after injection)	*In vitro*: γ-H2A RT ↑ RT + AGuIX® ↑↑ *In vivo* Survival RT ↑ RT + AGuIX® ↑↑	36
**Melanoma**	C57BL/6 mice	IVIS	ICB	Left striatum	B16	200 untreated + 1,800 disabled [pre-irradiated by 100 Gy (several hours before implantation)/1 culture medium	Philips RT100 X-ray	8	15/1	7.6	IT: tumor vaccine granulocyte-macrophage colony-stimulating factor	Median Survival RT↑ RT + IT ↑↑ Tumor volume RT ↓ RT + IT ↓↓	33
**Melanoma**	C57BL/6 mice	IVIS	ICB	Left striatum	B16-F10-luc2	250 B16-F10-luc2 + 5000 disabled B16 (100 Gy)/1 culture medium	Philips RT100 X-ray	8	15/1 18.75/1 22.5/1	7.6	IT	Median Survival 15 Gy ↑ 15 Gy + IT ↑↑ 18.75 Gy ↑ 18.75 Gy + IT ↑↑ 22.5 Gy ↑↑↑ 22.5 Gy + IT ↑↑↑	34
**Ehrlich Ascites tumor**	Female B6D2F1 mice	NA	ICB	Temporal hemisphere	Ehrlich ascites tumor (EHR2)	1.5×10^5^/30	Stabilipan (Siemens, Munich, Germany)	3	10/1	4.7	CTR: etoposide + dexrazoxane	Median Survival (vs RT) 34 mg/kg etoposide + 125 mg/kg dexrazoxane + RT ↑ 90 mg/kg etoposide + 125 mg/kg dexrazoxane + RT ↑ ↑	50
**Breast Cancer**	6-8w Female athymic nude mice	PET/CT 3D MRI	ICB	Right frontal lobe	BT474-Br-M3	4×10^5^/NA	Gamma Cell irradiator	8	10/ 5	NA	TT: Her2 Single-domain antibody fragments	synergistic effect	49
**Breast Cancer**	Athymic nude mice	IVIS	ICB	Left hemisphere	MDA-MB-231	1×10^5^/1	Philips RT100 X-ray generator (Amsterdam, The Netherlands) operating at 100 kVp with a 1.7 mm Al filter.	NA	15/1	NA	Nano: INP (iodine nanoparticles) i.v. (1 d before radiation)	Median Survival RT ↑ INP + RT ↑ Tumor size RT ↓ INP + RT ↓↓	38
**Breast Cancer**	6w Female BALB/c nude mice	IVIS	ICB	Right striatum	MDA-231-Br	2×10^4^/2	ELEKTA Irradiator (Precision X-Ray, UK)	5、17	10/2	NA	Leucine-rich repeat-containing protein 31 (LRRC31)	LRRC31 is a major DNA repair suppressor that can be targeted for cancer radiosensitization therapy.	37
**Breast Cancer**	6w Female athymic nude mice	NA	ICB	Left striatum	MDA-MB-435	1×10^5^/5	IBL 437C blood Irradiator	15	10/1	2.3	TT: c-Met tyrosine kinase inhibitor	Survival RT ↑ RT + inhibit c-Met ↑↑ Tumor volume RT ↓ RT + inhibit c-Met ↓↓ apoptosis RT ↑ RT +c-Met depletion ↑↑	44
**Breast Cancer**	Female athymic nude-Foxn1 nu mice	MRI	ICA	NA	MDA-MB-435-Br1	1×10^6^/100	NA	27	16.5/3	240 Monitor Units (MU/min)	CTR: Temozolomide	*In vivo* Survival RT + TMZ↑ vs. RT weight athymic mice— NOD/SCID mice ↓↓	55
**Breast Cancer**	12 w C57BL/6J 8w FVB/n mice	MRI	ICD	NA	99LN-BrM2 TS1-BrM	6×10^4^ 99LN-BrM 1×10^5^ 99LN-BrM 3×10^4^ TS1-BrM	SARRP	(About 3W) MRI indicates the brain metastases	10/5	5.2 cGy/s	IT: anti-PD-1	the combination of WBRT with Anti PD-1 has been demonstrated to exert a synergistic anti-tumor effect.	31
**Breast Cancer**	R2G2 SCID mice	bioluminescence imaging (BLI)	1. ICB 2. ICD	1. Brain at a depth of 4 mm.	SKBrM3 231-BrM	1.SKBrM3: 2×10^4^/5 231-BrM: 2×10^4^/5 2. SKBrM3: 1×10^5^ 231-BrM: 2×10^5^	X-Ray XRAD 320 Orthovoltage X-ray Unit with custom-made collimators (<5 mm diameter)	28 (When BLI reached 1 × 10^6^)	40/4	NA	A thermal radiofrequency electromagnetic field (1 day after tumor implantation.)	Survival: RT ↑ RT + BCF ↑↑	111
**Breast Cancer**	8-12w 1.NOD-SCID IL2Rγnull (NSG) Female mice 2.Female C57BL/ 6 mice	IVIS	ICD	NA	1.JmT1BR3-GFP-luciferase 2.E0711-GFP-Luciferase	1.2.5×10^5^ 2.5×10^4^	Precision X-Ray X-Rad 225Cx Micro IGRT and SmART Systems	7	1. 10/1 2. 35/1 10/1	4.8-5.8	Topiramate	Radiation-induced brain edema may be reduced by blocking AQP4	127

**Table 3:** Relevant Parameters of WBRT Research in the AM-BM

Cancer	Species	Injection method	Injection site	Cell line	Cell number/volume (cells/μl)	Irradiator	Irradiation time (days)	Dose/Fraction (Gy/F)	Dose rate (Gy/min)	Effect (WBRT vs control)	Ref.
**NSCLC**	7w Female athymic nude mice	ICB	Left striatum	PC14PE6 H460	1.0×10^4^/5	Blood irradiator	14	15/1	2.3	Survival 24/22 days —	75
**NSCLC**	6-8w Female athymic nude mice	ICD	NA	H2030-BrM	5×10^4^/100	SARRP (X-Strahl Ltd, Camberley, UK)	After the detection of metastatic lesions by MRI	10/5 10/1	5.2 cGy/s 220kV 13mA	Effects of WBRT on different TAM populations in BrM	32
**Breast Cancer**	5-7w Female athymic nude mice	1. ICD 2. ICB	Right hemisphere	MDA-MB-231-BR	1.1.75×10^5^/NA 2.1×10^6^/NA	Pentak X-irradiator	1)14-23 2)14	1)30/10 2)15/1	2.53 300 kV 10 mA	30 Gy/10F significantly reduced intracranial metastases, while 15 Gy/1F was more beneficial for median survival	76
**Breast Cancer**	6-8w Female nu/nu mice	ICD	NA	MDA-MB-231-BR	1.5×10^5^/100	modified GE eXplore CT 120	26	8/1 16/1 24/1	NA	Mean fractional growth of brain metastases↓ DSB ↑ γ-H2AX ↑ Cell density ↑ nuclear size ↑	79
**Breast Cancer**	6-7w Female BALB/c nu/nu mice	ICD	NA	MDA-MB-231-BR	2×10^5^/50	X-RAD 320 orthovoltage irradiator	MRI indicates the brain metastases (about 3W)	12/3	2.33	Tumor volume ↓ Apoptosis ↑	52
**Breast Cancer**	10-12w Female C57BL6/J mice	ICD	NA	99LN-BrM	6×10^4^/NA	SARRP	After the detection of metastatic lesions by MRI	10/1	5.2 cGy/s 220 kV 13 mA	WBRT (10 Gy/1F) reduced tumor volume with no significant difference in survival CC3 — Ki67 ↓	53
**Breast Cancer**	6-8w Female nude mice	ICD	NA	MDA-MB-231-BR-HER2	1×10^5^/100	integrated micro-computed tomography (CT)/RT system	24 and 25	20/2	0.12 ± 0.01	Mean tumor volume ↓ the number of tumors — total tumor volume — γ-H2AX ↑(DSB)	54
**Breast Cancer**	8 w Female nu/nu mice	ICD	NA	MDA-231-Br	1.75×10^5^/100	Xrad-225Cx irradiator (PXi, Cyceron platform)	18	12/3	3.3 225 kV X-rays	αVCAM-1 showed better tumor growth inhibition than WBRT	87
**1. NSCLC** **2. Breast cancer**	4-6 w athymic nu/ nu (Harlan) C57BL/6 mice	1. ICD 2. ICB	1. NA 2. Right frontal cortex	1.H2030-BrM 2.E0771-BrM3	1. 1×10^5^/100 2.2.5×10^4^/2	The irradiator Mark I 30 A	1.7 (30 Gy/10F) 2.3 (30 Gy/10F)	30/10 16.5/3 10/3	NA	*In vivo* OS — *in vitro* S100A9 ↑; RAGE ↑; NF-kB↑; JunB↑; oncosphere CD55+↑	88

**Table 4:** Relevant Parameters of the SRS Research in the AM-BM

Cancer	Species	Monitoring intracranial tumor formation	Injection method	Injection site	Cell line	Cell number/volume (cells/µl)	Irradiator	Irradiation time (days)	Dose/Fraction (Gy/F)	Dose rate (Gy/min)	Combination Therapy	Effect	Ref.
**NSCLC**	7w Female C57BL/6 mice	IVIS	ICB	2 mm right lateral and 1 mm posterior of the bregma. The injection depth was adjusted to 3 mm.	LLC	2×10^5^/3	Leksell Gamma Knife (LGK)	10	2/1 12.4/2	NA	Nano: HVGGSSV-chitoPEGAcHIS-SP600125 (HVSP-NP)	Tumor size IR ↓ IR+HVSP-NP ↓↓ Survival IR ↑ IR+HVSP-NP ↑↑ p-JAK (vs IR) IR+HVSP-NP ↓ γH2AX IR+HVSP-NP ↓ Cleaved Caspase3 IR+HVSP-NP ↑	35
**Melanoma**	4-6w Female C57BL/6J mice	Volumetric computerized tomography	ICB	Right striatum	B16-F10	5×10^2^/ 5	Gamma Knife Model 4C model (Stockholm, Sweden)	4	15/1	2.51	Thymoquinone (TQ) same day as tumor implantation,	*In vivo* Median survival time GK ↑ GK+TQ ↑	42
**Melanoma**	7w male C57BL6 mice	MRI; H&E staining; fluorescent microscopy for GFP	ICB	1 mm anterior and 2 mm lateral to the bregma; lowered to 2.5 mm depth from the surface of the brain	B16-F10	300/1	SARRP	11	18/1	NA	NA	Tumor volumes IR ↓ Survival IR ↑	73
**Breast Cancer**	Fischer 344 rats	NA	ICB	1.8 mm anterior to the bregma and 2 mm to the right of the sagittal suture to a depth of 4 mm below the surface of the skull	MADB106	5×10^3^/5	201-source ^60^Co Leksell gamma knife	5	32/1	NA	IT:2×10^6^ transduced tumor cells vaccination (GM-CSF vaccine or the IL-4 vaccine) 3days	Median survival (vs control) IR ↑ IR+IT ↑↑ Infiltration of CD11b/c+ cells SRS ↑ IT ↑ IT + SRS ↑ CD4+cells IT ↑ IT + SRS ↑ αβTCR+ CD8+, and CD28+ cells IT ↑ IT + SRS ↑	72

**Table 5:** Relevant Parameters of the PCI Research in the AM-BM

Cancer	Species	Monitoring intracranial tumor formation	Injection method	Cell line	Cell number/ volume (cells/µl)	Irradiator	Irradiation time (days)	Dose/Fraction (Gy/F)	Dose rate (Gy/min)	Effect (vs control)	Ref.
**Breast** **Cancer**	6-8w Female nude mice	MRI	ICA	MDA-MB-231-BR-HER2	1.75×10^5^/100	custom micro-irradiation system	1 and 2	20/2	0.13±0.01	Tumor volume ↓ Tumor number ↓ Non-proliferative cancer cells — (the iron label)	78
**Breast** **Cancer**	3-5w Female SCID/Beige mice	NA	TVI	MDA-IBC3 (HER2-neu-overexpressing)	5×10^5^/NA	NA	2 (before cells were injected) 5, 21, or 42	4/1	NA	The rates of brain metastasis↓ (5 days)	57
**Breast** **Cancer**	3-5w Female immunocompromised SCID/Beige mice (Harlan, USA)	IVIS	TVI	MDA-IBC3	5×10^5^/200	X-RAD 225Cx small-animal irradiator (PRECISION X-RAY, North Branford, CT, USA)	2 (before tumor-cell injection) 5 days, 3 weeks, or 6 weeks	4/1	3.2	The rates of brain metastasis↓ (5 days)	56
**Breast** **Cancer**	6-8 w Female BALB/c mice	MRI	ICA	4T1‐BR5	2×10^4^/100	In‐house custom micro‐irradiation system (140 kVp, 50 kW) with on‐board image guidance	7 (before cell delivery)	10/1	NA	Tumor volume and number ↑	51
**NSCLC**	6-8 w male BALB/c nude mice	IVIS	ICD	A549-F3	2×10^5^/100	Rad Source Technologies Inc., Suwanee, GA	7 (before cell delivery)	6/2	NA	PCI activates microglia, reduces the localization ability of NSCLC brain metastasis cells	134

**Table 6:** Relevant Parameters of the RT/IMRT in the AM-BM (RT, IMRT, etc.)

Cancer	Species	Injection method	Injection site	Cell line	Cells number/volume (cells/µl)	Irradiator	Irradiation time (days)	Dose/fraction (Gy/F)	Dose rate (Gy/min)	Irradiation method	Combination Therapy	Effect	Ref.
**Melanoma**	10w nude rats	ICB	Right caudate nucleus	MRA 27	1×10^6^/NA	linear accelerator (Siemens Medical Systems, Concord, CA).	12-14	15/3	NA	RT	Boron neutron capture therapy BNCT (BNCT i.v.) BPA (500 mg/kg) containing an equivalent amount of 10B (27 mg B/kg).	Survival: RT ↑; Neutron + RT ↑↑; BPA + BNCT+ RT ↑↑↑; BPA + BNCT +RT ↑↑↑	48
**Melanoma**	6w Female nude mice	ICB	1 mm anterior to bregma, -1 mm lateral, and -3 mm in deep of the cortex surface	B16-F10	1×10^4^/NA	Faxitron CP-160 irradiator	NA	14/7	1.0 Gy/min	IMRT	5-aminolevulinic acid(5-ALA) 200 mg/kg 4 h before X-ray irradiation	*In vivo* Tumor size RT ↓ 5-ALA+RT ↓↓ *In vitro* γH2AX RT ↑ 5-ALA+RT ↑↑	101
**Breast Cancer**	6w Female BALB/c nude mice	ICB	The brain 2 mm posterior, 1.5 mm right lateral, and 3.5 mm deep from the bregma	MDA-MB-231(Br)	2×10^5^/2 PBS	X‐Rad 320 (Precision X‐Ray, North Branford, CT)	14	15/5	NA	RT	Metformin (300 mg/kg/d) 1 week after Tumor implantation	Metformin increases the concentration of lactate in MDA-MB‐231-Br cells by suppressing MCT4 protein, thereby enhancing the antitumor effect of RT	65
**Breast Cancer**	Female Berlin-Druckery IX (BD-IX) rats	ICB	Left striatum	ENU1564	1×10^3^/NA	SARRP irradiator (Xstrahl, Camberley, UK)	20	25 /1	NA 225 kV x-rays	IMRT	NA	Tumor volumes RT ↓	112
**Breast Cancer**	Female immunodeficient nude mice	ICB	Right hemisphere	MDA-MB-231-BR	1×10^5^/5 PBS	small-animal radiation research platform (Xstrahl, Camberley, England)	NA	10 /1	2.42 Gy/min 225 kV [peak]	IMRT low-linear energy transfer radiation	Microbubble	Tumor volumes: RT ↓RT+ O_2_ MBs ↓; RT+O_2_ MBs + US ↓↓ Median Survival: RT ↑; RT+ O_2_ MBs ↑; RT+O_2_ MBs + US ↑↑	86
**Breast Cancer**	4-6w Female nude mice	ICB	Caudate nucleus	MDA-MB-231-BR	1×10^6^/5	Pantak irradiator	3	5 /1	2.28 Gy/min	RT	TT: vorinostat (75 mg/kg)	*In vivo* Tumor volume RT ↓ RT +vorinostat↓↓ Survival RT ↑ RT +vorinostat↑↑ *In vitro* γH2AX RT ↑ RT +vorinostat↑↑ Mitotic catastrophe RT ↑ RT +vorinostat↑↑	47

**Table 7:** A Comprehensive and Scientific Template for Reporting Experiments Involving Animal Models of Brain Metastasis Radiotherapy

Parameter	Notes on Reporting
Study design	
Assessment time-points post-irradiation	in Weeks
Serial assessment of >=1 parameter completed?	Yes/No
Combination therapy	Immunotherapy, Chemotherapy, Targeted therapy
Sequence of combination therapy	—
Tumor parameters	
Tumor type	Lung cancer, Breast cancer, Melanoma
Injection method	Orthotopic injection, Intracardiac injection, Carotid artery injection, Tail vein injection
Time of cell injection	in Weeks
Number of tumor cells	—
Cell line	A549, LLC, 4T1, B16-F10
Injection site	Frontal lobe, Striatum
Tumor size	
Radionuclide imaging	Yes/No
Magnetic resonance imaging (MRI)	Yes/No
Bioluminescence imaging (BLI)	Yes/No
CT/PET-CT	Yes/No
Animal character	
Animal species	Mouse or Rat
Animal strain	C57BL/6, BLAB/c, SCID, SD
Sex	Male or Female
Gene modifications/spontaneous mutations	—
Irradiation	
Irradiation time (after/before irradiation)	in Weeks
Target volume	Whole Body, Head, Whole Brain, or Partial Brain
Form of ionizing radiation	X-rays, Gamma rays, Electrons, Heavy ions
Energy of radiation	in kV or MV
Dose rate	in Gy/min
Field arrangement	—
Radiation device used	Brand & Model
Dose fractionation	
Total physical irradiation dose	in Gray
Total fractions	—
Duration over which irradiation was given	in Days
Functional analyses	
Blood-brain barrier permeability	Yes/No
Animal weight	Yes/No
Tumor size	Yes/No
Radiotherapy-related markers	Yes/No
Invasive hemodynamics	Yes/No
Tumor markers	Yes/No
Radiography	Yes/No

## Abbreviations

BMs: brain metastases
AM-BM: animal model of brain metastases
WBRT: whole brain radiation therapy
SRS: stereotactic radiosurgery
PCI: prophylactic cranial irradiation
RT: Radiotherapy
IMRT: Intensity-modulated radiation therapy
ICB: intracerebral injection
ICD: intracardiac injection
ICA: intracarotid artery injection
IV: Intravenous inoculations
TVI: tail vein injection
PDX: Patient-derived tumor xenograft
MRI: magnetic resonance imaging
BBB: the blood-brain barrier
L-Q: The linear-quadratic
BED: the Biologically effective dose
D: total Dose
d: single dose
TAM-MDM: monocyte-derived macrophages
TAM-MDM: monocyte-derived macrophages
BTB: blood-tumor barrier
FUS: focused ultrasound
TMZ: temozolomide
NSCLC: non-small cell lung cancer
AMONs: anti-MGMT morpholino oligonucleotides
EGFR: Epidermal growth factor receptor
HER2: growth factor receptor type 2
HDACs: Histone deacetylases
ES: Endostar
ICI: immune checkpoint inhibitors
ISV: *in situ* vaccination
IVIS: *In vivo* Imaging System
NF-κB: nuclear factor kappa-light-chain-enhancer of Activated B cells signaling
FLASH-RT: FLASH radiotherapy
RAGE: receptor for advanced glycation end products
MBs: microbubbles
5-ALA: 5-aminolevulinic acid
PpIX: protoporphyrin IX
TQ: Thymoquinone
GRM1: metabotropic glutamate receptor 1
SARRP: the Small Animal Radiation Research Platform
LLC: Lewis lung cancer
IT: immunotherapy
TT: targeted therapy
CTR: Chemotherapy
GK: Gamma Knife
NA: Not available
